# A Role for Oxytocin in the Etiology and Treatment of Schizophrenia

**DOI:** 10.3389/fendo.2015.00090

**Published:** 2015-06-03

**Authors:** Megan Elizabeth Rich, Heather Kingsley Caldwell

**Affiliations:** ^1^Laboratory of Neuroendocrinology and Behavior, Department of Biological Sciences, The School of Biomedical Sciences, Kent State University, Kent, OH, USA

**Keywords:** dopamine, early life stress, glutamate, social cognition, sensorimotor gating

## Abstract

Schizophrenia is a chronic debilitating neuropsychiatric disorder estimated to affect 51 million people worldwide. Several symptom domains characterize schizophrenia, including negative symptoms, such as social withdrawal and anhedonia, cognitive impairments, such as disorganized thinking and impaired memory, and positive symptoms, such as hallucinations and delusions. While schizophrenia is a complex neuropsychiatric disorder with no single “cause,” there is evidence that the oxytocin (Oxt) system may be dysregulated in some individuals. Further, treatment with intranasal Oxt reduces some of the heterogeneous symptoms associated with schizophrenia. Since Oxt is known for its modulatory effects on a variety of social and non-social behaviors, it is perhaps not surprising that it may contribute to some aspects of schizophrenia and could also be a useful therapeutic agent. In this review, we highlight what is known about Oxt’s contributions to schizophrenia and schizophrenia-related behaviors and discuss its potential as a therapeutic agent.

## Introduction

Schizophrenia, a chronic and debilitating neuropsychiatric disorder, affects 1% of the population worldwide (1). According to the fifth edition of the Diagnostic and Statistical Manual of Mental Disorders, schizophrenia is characterized by a combination of negative symptoms, cognitive dysfunction, and positive symptoms (2). Negative symptoms of schizophrenia include deficits in social behaviors such as social withdrawal, anhedonia, and flattened affect. Cognitive impairments include disorganized thinking and impaired executive function, working memory, and attention (3, 4). Lastly, the positive symptoms of schizophrenia include hallucinations, paranoid delusions, and disorganized speech. Unfortunately, while current antipsychotic medications are effective at ameliorating the positive symptoms, they are not very effective at treating the negative symptoms and cognitive dysfunction associated with schizophrenia, which tend to be more pervasive and persistent (5–7).

Current antipsychotic therapies are based on the dopamine hypothesis of schizophrenia, which proposes that increases in dopamine transmission in the mesolimbic dopamine pathway, and decreases in its activity in the prefrontal cortex contribute to many of the observed symptoms (8–10). As such, typical antipsychotics are dopamine 2 (D2) receptor antagonists, which only reduce positive symptom severity. Atypical antipsychotics on the other hand are reported to alleviate the positive symptoms as well as some of the negative symptoms associated with schizophrenia. These medications inhibit the serotonin 2A receptor (5-HT_2A_), and to a lesser extent D_2_ receptors and other neurotransmitter systems associated with schizophrenia, such as the adrenergic and cholinergic systems (11). However, two large clinical studies, the Clinical Antipsychotic Trials of Intervention and Effectiveness (CATIE) and the Cost Utility of the Latest Antipsychotic Drugs in Schizophrenia Study (CUtLASS) found no significant difference between the ability of typical and atypical antipsychotics to reduce the negative symptoms and cognitive dysfunction of schizophrenic patients (12–15). Thus, it is important to better understand the neurochemistry of the negative symptoms and cognitive dysfunction as they often precede the onset of the positive symptoms and act as better predictors of therapeutic outcome (16, 17). Due to the various combinations of symptoms and the wide range of symptom severity, diagnosis and treatment of schizophrenia are difficult; making it extremely important to elucidate which neurological factors may contribute to schizophrenia as well as identify treatments that can effectively lessen symptom severity.

## Schizophrenia

**Figure 1:**
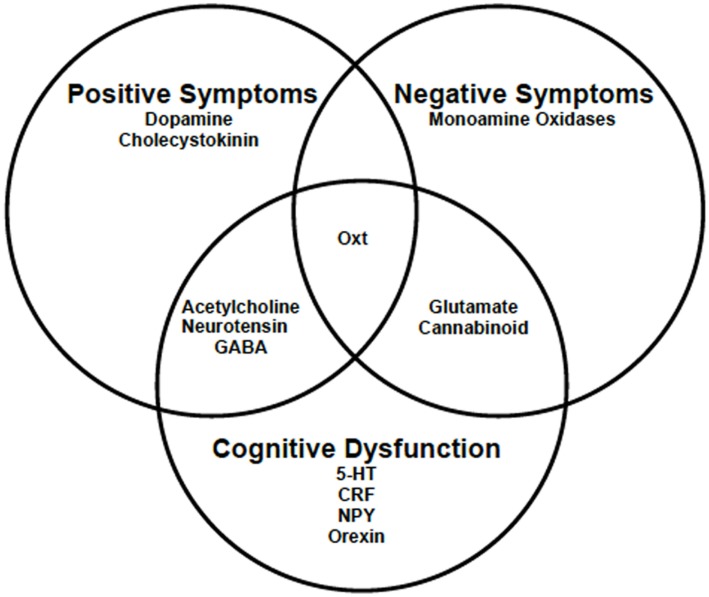
**The symptom domains of schizophrenia and the neurotransmitter and neuropeptide systems known to play a role**. Research suggests that Oxt may play a role in all three symptom domains associated with schizophrenia. 5-HT, serotonin; CRF, corticotropin-releasing factor; GABA, gamma aminobutyric acid; NPY, neuropeptide y; Oxt, oxytocin.

Schizophrenia is a heterogeneous group of disorders, and as such no single gene can explain its pathophysiology. Hence, it is not surprising that several neurotransmitter and neuropeptide systems, beyond dopamine, have been implicated in its symptomology (Figure 1) [for review, see Ref. (8, 18–20)]. In addition to the dopamine hypothesis, there is the glutamate hypothesis, which supposes that it is the hypofunctioning of *N*-methyl-d-aspartate (NMDA) receptors that contribute to the negative symptoms and cognitive impairments associated with schizophrenia (19). Researchers studying the cholinergic and gamma aminobutyric acid (GABA) systems have found that these neurotransmitter systems may also play a role in both the psychotic and cognitive deficits found in schizophrenia patients (20, 21); while serotonin (5-HT) is mainly implicated in only the cognitive dysfunction associated with schizophrenia (22–24). Cannabinoids and monoamine oxidase, which modulate some of these neurotransmitter systems, also appear to also play a role in the negative symptoms and cognitive deficits (25, 26). Since many neuropeptides are often co-released with these neurotransmitters, they likely have a role to play as well. Some of these neuropeptides are neurotensin, cholecystokinin, corticotropin-releasing factor, neuropeptide y, and orexin (18). One neuropeptide that interacts with several of the aforementioned neurotransmitter and neuropeptide systems is the nonapeptide oxytocin (Oxt). Further, there is evidence that Oxt may be important to the etiology, symptom severity, and potential treatment of schizophrenia. First, in schizophrenic patients, there are reports of disruptions in the Oxt system that are affected by treatment with antipsychotics (27, 28). Second, treatment with Oxt as an adjunctive therapy is known to lessen symptom severity in some (29, 30). Third, animal models of schizophrenia suggest that Oxt may be involved in all three symptom domains (31–35).

## Oxytocin

Oxt is a nine amino acid peptide hormone, synthesized primarily in neurons of the hypothalamic supraoptic (SON) and paraventricular (PVN) nuclei. To date, a single seven-transmembrane G-protein coupled receptor, known as the Oxt receptor (Oxtr), is thought to mediate the actions of Oxt; although Oxt can also bind to the vasopressin (Avp) 1a and 1b receptors [for review, see Ref. (36)]. The Oxt system is involved in regulating a variety of behaviors [for review, see Ref. (37)] and implicated in aspects of learning and memory, such as spatial and non-spatial memory (38–41). However, more commonly, Oxt is known for its importance to the neuromodulation of social behaviors such as social memory and social recognition, affiliative behaviors, and aggression [for review, see Ref. (37, 42)]. Social behaviors are evolutionarily important because they reduce stress and anxiety (43, 44) and in humans, Oxt facilitates prosocial behaviors and increases feelings of trust and empathy (45–47). Given the effects of Oxt on social behaviors, it is perhaps not surprising that research has focused on the role of Oxt in neuropsychiatric disorders that are characterized by disruptions in social functioning.

## Abnormalities in the Oxytocin System

Due to the negative symptoms associated with schizophrenia, and the effects of Oxt on prosocial behaviors, researchers hypothesize that Oxt dysregulation may contribute to the etiology and symptom severity of schizophrenia (29, 30). This hypothesis is supported by studies indicating that disruptions in the Oxt system are linked to the pathophysiology of schizophrenia (28, 48, 49). Altered levels of Oxt are reported in patients with schizophrenia (50, 51). However, the data are conflicting with some studies reporting an increase in Oxt and the Oxt carrier protein neurophysin I (50, 51) and another reporting no change in Oxt levels in cerebral spinal fluid (CSF) (52). However, patients with higher plasma levels of Oxt have less severe positive symptoms and exhibit fewer social deficits (53, 54).

Recently it has been reported that single nucleotide polymorphisms (SNPs) of the *OXT* and *OXTR* genes may contribute to symptom severity and treatment efficacy in schizophrenic patients (55–57). SNPs of the *OXTR* gene are associated with the severity of symptoms and the improvement of the positive symptoms of schizophrenia following treatment with antipsychotics (27, 28). Additionally, post-mortem analysis of brain tissue from unmedicated schizophrenia patients found altered neurophysin immunoreactivity (ir) in the PVN, internal palladium, and substantia nigra (58). Most recently, in patients with schizophrenia and polydipsia, decreases in plasma Oxt were found to correlate with the ability to correctly identify facial emotions (48) as well as malformations in brain areas that mediate neuroendocrine responses such as the anterior lateral hippocampus and amygdala (Amg) (59). Together, these data suggest that alterations in the function of the Oxt system may underlie all three symptom domains associated with schizophrenia. Given the dysregulation of the Oxt system in patients with schizophrenia, Oxt has been studied as a candidate for use as a therapeutic.

Human studies suggest that Oxt may have antipsychotic properties [for review, see Ref. (60, 61)]. Previous work found that injections of Oxt reduce the symptoms of psychosis and anhedonia in patients with schizophrenia (62, 63). Due to the ease of delivery, researchers are now utilizing intranasal administration of Oxt. It should be noted that there is an ongoing debate in the field on whether or not intranasal administration of Oxt is able to cross the blood–brain barrier, but there is evidence that intranasal administration increases Oxt concentrations in CSF in humans and animal models (64–66). In healthy patients, intranasal Oxt increases holistic processing, divergent thinking, and creative cognition (67), and studies in patients diagnosed with schizophrenia report that intranasal Oxt can be beneficial. Specifically, intranasal Oxt can facilitate social cognition (30, 68–70) and alleviate some of the cognitive deficits and positive symptoms in patients with schizophrenia (69). Yet, intranasal Oxt may be most effective as an adjunctive therapy to already prescribed antipsychotics, where chronic treatment is able to ameliorate some of the negative symptoms and the cognitive deficits, as well as the positive symptoms (30, 71, 72). While this research suggests that Oxt treatment has the potential to improve symptoms in all three domains, where in the brain and how these effects are mediated remains unknown. Animal models for schizophrenia are being used to determine where and how Oxt treatment may improve symptoms associated with schizophrenia.

## Oxytocin in Humans and Animal Models

There are inherent challenges when studying a multifaceted disorder such as schizophrenia. Therefore, reliable animal models are necessary to understand and develop viable treatments. A good animal model must have phenotypic overlaps with either a behavior or a molecular characteristic of the disease. In humans, schizophrenia is characterized by several endophenotypes, including impairments in social behaviors such as emotion processing, social perception, attributional bias, and theory of mind [for review, see Ref. (73)]. Schizophrenic patients also have deficits in sensorimotor gating, as measured by prepulse inhibition (PPI) of the acoustic startle reflex [for review, see Ref. (74)], and cognitive deficits in verbal and visual memory, and impaired cognitive flexibility [for review, see Ref. (75)]. There are also neuromotor abnormalities such as dysmetria, eye tracking dysfunctions, and saccadic eye movements, which are typically associated with the positive symptoms of schizophrenia [for review, see Ref. (76–78)], as well as structural abnormalities in total brain volume and the volume of specific brain regions including, but not limited to, the hippocampus, the lateral ventricles, and the prefrontal cortex [for review, see Ref. (77, 79)] Co-morbid anxiety disorders are found in 38% of schizophrenia patients, and studies have reported increases in violent behaviors in schizophrenic patients (80–82). While changes in anxiety-like behavior and aggression have not been proposed as animal models for schizophrenia, several existing animal models of schizophrenia result in altered anxiety-like and aggressive behavior (83–91). Further, atypical antipsychotics have been found to reduce anxiety and reverse psychosis-induced aggression in patients with schizophrenia (92–96). Therefore, the examination of anxiety-like and aggressive behaviors seems warranted.

Currently, over 20 animal models are being used to assess the heterogeneous symptoms associated with schizophrenia (97). To study the specific contributions of the Oxt system, several models have been developed. The first utilizes perinatal stress, since research in humans suggests that exposure to adverse environmental conditions during perinatal development increases the risk for schizophrenia (98). Stress during the perinatal period is known to induce the behavioral and molecular characteristics of schizophrenia and is commonly used to model the negative symptoms of schizophrenia (85, 86, 99). The second employs the pharmacological disruption of the dopaminergic and glutamatergic systems, since the pathophysiology of schizophrenia suggests that there is dysfunction in both of these systems. Treatment with amphetamine (AMP), an indirect dopamine agonist, or phencyclidine (PCP), an NMDA receptor antagonist, induces hyperlocomotor activity, which corresponds with the positive symptoms of schizophrenia (100–103). Further, PCP treatment induces both negative symptoms and cognitive dysfunction, such as social withdrawal (104–106), impaired PPI (107), and cognitive deficits (108). The third uses gene knockout, since schizophrenia is a genetic disorder with high levels of heritability. Recently, it has been reported that genetic mutations in Oxt genes are associated with schizophrenia (55). It is for this reason that mice with genetic disruptions of their Oxt systems, such as Oxt and Oxtr knockout mice (Oxt−/− and Oxtr−/−, respectively) have been used to determine their potential contributions to the symptoms associated with schizophrenia. While no single model is sufficient to encompass all of the heterogeneous symptoms of schizophrenia, together these models can help us to better understand the role that Oxt may play in schizophrenia. It should be noted that several of these models are not specific to schizophrenia, and the data are relevent for other neuropsychiatric disorders (109). Currently, all of the aforementioned models are being used to study the relationship of Oxt to the negative symptoms of schizophrenia, and while some have been used to study the cognitive deficits and positive symptoms, more research is needed.

### Deficits in social behaviors

Oxt has a well-characterized role in the neural regulation of social behaviors [for reviews, see Ref. (37, 42, 63, 110, 111)]. It is therefore not surprising that Oxt is studied for its potential contributions to the modulation of the negative symptoms of schizophrenia (Table 1). This section is broken up according to the approaches described in the previous section, as there is far more data on the contributions of Oxt to deficits in social behaviors than there are for the other symptoms associated with schizophrenia.

**Table 1:** **Oxt and social deficits associated with schizophrenia**.

Animal model	Species	Main findings	Author	Relevant findings in humans	Author
Early life stress	Mouse	↓ Intermale aggression ↑ Maternal aggression ↑ Oxt-ir in PVN in males ↓ Oxt-ir in PVN in females	Tsuda et al. (84) Veenema et al. (83)	↓ Oxt in CSF in adult females with history of childhood abuse ↓ Plasma Oxt in adult males exposed to early life stress ↑ Plasma Oxt in adult females exposed to trauma in childhood following psychosocial challenge ↓ Plasma Oxt in children exposed to early neglect following interactions with their mother compared to controls APO treatment ↓ Plasma neurophysin in patients with schizophrenia compared to controls Higher plasma Oxt levels in patients with schizophrenia results increased social cognition and fewer negative symptoms ↓ Plasma Oxt in male patients with schizophrenia and increased negative symptoms Lower CSF Oxt in male schizophrenic patients corresponds with increased negative symptoms ↓ Plasma Oxt in patients with schizophrenia after trust exercise compared to controls	Heim et al. (112) Opacka-Juffry and Mohiyeddini (113) Pierrehumbert et al. (114) Fries et al. (115) Legros et al. (116) Goldman et al. (48) Rubin et al. (53) Rubin et al. (54) Strauss et al. (117) Strauss et al. (118) Jobst et al. (119) Sasayama (120) Keri et al. (121)
	Rat	↑ Oxt-ir with prolonged separation in males	Oreland et al. (122)		
		↑ Oxtr binding in MPOA and VMH ↓ Oxtr binding LS, AG, and CP	Lukas et al. (123)		
	Mandarin Vole	↑ Oxt-ir until PND8 in PVN and PND4 in SON after social isolation ↓ Oxt-ir until PND 14 in PVN after paternal deprivation	Wang et al. (124)		
Prenatal stress	Rat	↑ Aggression and Anxiety ↓ Social recognition and social interaction ↓ Oxt-ir in PVN	de Souza et al. (125)		
		↓ Social recognition and social interaction ↑ Oxtr binding CeA ↓ Oxt mRNA in PVN Oxt administered to CeA reversed social deficits	Lee et al. (126)		
Dopamine agonist	Prairie Vole	Subchronic AMP treatment ↓ Pair bond formation ↓ Oxtr-ir in mPFC/PLC Oxt administered to PLC restores pair bond formation	Young et al. (127)		
NMDA antagonist	Rat	Chronic PCP treatment ↓ Social interaction ↓ Oxt mRNA in PVN ↑ Oxtr binding CeA Oxt administered to CeA restores social deficits	Lee et al. (105)		
Dysregulation of the Oxt system – Oxt and Oxtr knockout mice	Mouse	↓ Social memory and Social recognition in Oxt and Oxtr−/− mice	Ferguson et al. (128) Nishimori et al. (31) Takayanagi et al. (32)		
		Oxt administration to Amg restores deficits in social recognition in Oxt−/− mice	Winslow and Insel (33)		
		↑ Social withdrawal in Oxtr−/− mice in visible burrow paradigm ↑ Social withdrawal in Oxtr−/− mice in three-chamber test	Pobble et al. (129, 130)		
		↑ Intermale Aggression Oxt and Oxtr−/− mice	Winslow et al. (131) Dhakar et al. (90)		
		↓ Maternal aggression Oxt−/− mice	Young et al. (91)		
		↓ Initiation Maternal Behavior Oxtr−/− mice and Oxtr FB/FB	Macbeth et al. (132) Rich et al. (133)		
		↓ Ultrasonic vocalization in Oxt−/− mice pups	Winslow et al. (131)		

### Perinatal stress

Research in humans has demonstrated that there is a positive correlation between perinatal exposure to a stressful environment and increased risk of schizophrenia (98). In rodents, maternal separation modifies aggressive behavior, and decreases social recognition, anxiety-like, and depression-like behaviors (85, 86, 99); with the effects of early life stress on aggression and Oxtr distribution being sex specific in both mice and rats. Following maternal separation, male mice exhibit decreases in aggression (83, 84) and increases in Oxt-ir in the PVN (84). However, in female mice, maternal separation results in increases in maternal aggression and decreases in Oxt-ir cells in the PVN (83). Similar to mice, in male Long Evans rats, early life stress results in decreases in intermale aggression, and in male Wistar rats, prolonged maternal separation results in increases in Oxt-ir in the Amg (122), increases in Oxtr binding in the medial pre-optic area (MPOA) and ventromedial hypothalamus (VMH), and decreases in Oxtr binding in the lateral septum (LS), agranular cortex, and caudate putamen (CP) in adulthood (123). Early life stress in female Wistar rats results in increases in aggression (134, 135). Data from another rodent species, mandarin voles, have shown that neonatal social isolation results in increases in Oxt-ir in the PVN until post natal day (PND) 8 and the SON until PND4 in both sexes (124). Further, in vole pups that have been isolated from their fathers there is a downregulation of Oxt-ir neurons until PND14, but these decreases do not persist (124).

In addition to maternal separation, prenatal stress can also cause behavioral effects in rodents that are reflective of symptoms of schizophrenia. Adult male rats subjected to prenatal stress and reared by stressed mothers display lower levels of aggression and social behaviors, and increases in anxiety-like behaviors (125, 126, 136). However, when non-stressed mothers rear pups that are exposed to stress during the prenatal period, the deficits in aggressive behaviors and increases in anxiety do not persist (125). Further, these effects appear to be due to Oxt, as an injection of Oxt into the central amygdala (CeA) is able to restore the social deficits exhibited by male rats subjected to prenatal stress (126). Male offspring raised by their prenatally stressed mothers also have reductions in Oxtr mRNA, fewer Oxt positive magnocellular neurons in the PVN, and increases in Oxtr binding in the CeA (125, 126). These morphological changes in Oxt system are not found when non-stressed dams raise the pups.

The behavioral differences observed between species, strain, and sex that result from stress during the perinatal period appear to be a result of alterations in the Oxt system. Many of the changes in the Oxt system are found within the neuronal network that mediates aggression: the MPOA, LS, anterior hypothalamus, VMH, medial amygdala (MeA), and bed nucleus of the stria terminalis (BNST) (137). There are also changes found in the Oxt system in the PVN, and it is known that stress can modulate aggression via the PVN (137). In males, perinatal stress results in decreases in aggression and increases in Oxt-ir and Oxtr binding (83, 84, 122, 134). However, in females, increases in aggression coincided with decreases in Oxt signaling (83, 134, 135). These sex differences in aggression and Oxt may be a result of estrogen-mediated sex differences in Oxtr regulation (138, 139).

Low levels of licking/grooming (LG) maternal behavior are associated with decreases in estrogen receptor-alpha (ERα) and Oxtr levels in the MPOA in female offspring (138, 139). Further, the interactions of estrogens and the Oxt system may result in changes to the dopamine system, as females reared by low LG dams have fewer dopamine neurons in the VTA (140). Research using dopamine agonists to model schizophrenia suggest that there are important interactions between the Oxt and dopaminergic systems to social cognition. Taken together, the data from perinatal stress models suggest that there can be long-lasting disruptions of Oxt neurochemistry, which may lead to impairments in behaviors that are similar to the negative symptoms of schizophrenia.

### Pharmacological disruption

Pathophysiological studies utilizing dopamine agonists and NMDA receptor antagonists have reaffirmed the importance of Oxt to social cognition in patients with schizophrenia. A study on drug addiction and social behaviors provides insight into the role of Oxt, dopamine, and social behaviors (127). Specifically, in prairie voles, repeated subchronic AMP exposure inhibits pair bond formation (127), decreases Oxtr-ir in the mPFC, and reduces Oxtr activation in the PLC; which is important for partner preference formation (127, 141). Additionally, Oxt direct infusion into the PLC is able to restore AMP-induced impairment in partner preference and alter dopamine levels in the nucleus accumbens (NAcc) (127). Administration of PCP induces social dysfunctions in animals that mimics the negative symptoms associated with schizophrenia [for review, Ref. see (142, 143)]. Oxt mRNA expression is reduced in the PVN of rats and Oxtr binding is increased in the CeA following chronic PCP treatment (105). Further, PCP-induced deficits in social interactions are increased by bilateral infusions of Oxt to the CeA (105). While these data suggest that the interaction of Oxt with both dopamine and glutamate is important for social behavior, the specific mechanisms that mediate these effects remain unclear.

Research on sex behavior in rats suggests that the dopaminergic and Oxt systems can modulate each other (144–146), and the Oxtr is located throughout the mesolimbic dopamine pathway (147, 148). Thus, researchers have hypothesized that Oxt and dopamine may work together to affect on how an individual perceives the salience of social cues [for review, see Ref. (149)]. However, the connection between these two systems and their role in schizophrenia remains murky. Likewise, the link between the Oxt and glutamate systems is also poorly understood. In rat SON preparations, application of both Oxt and Avp inhibits glutamate release (150). However, in cultured rat olfactory bulb neurons glutamate transmission is facilitated (151). More recently, it has been found that in the CeA, Oxt and glutamate are co-released from Oxt neurons (152). More research is still needed to determine how and where Oxt may interact with these neurotransmitter systems to affect social cognition in patients with schizophrenia.

### Genetic disruptions

**Figure 2:**
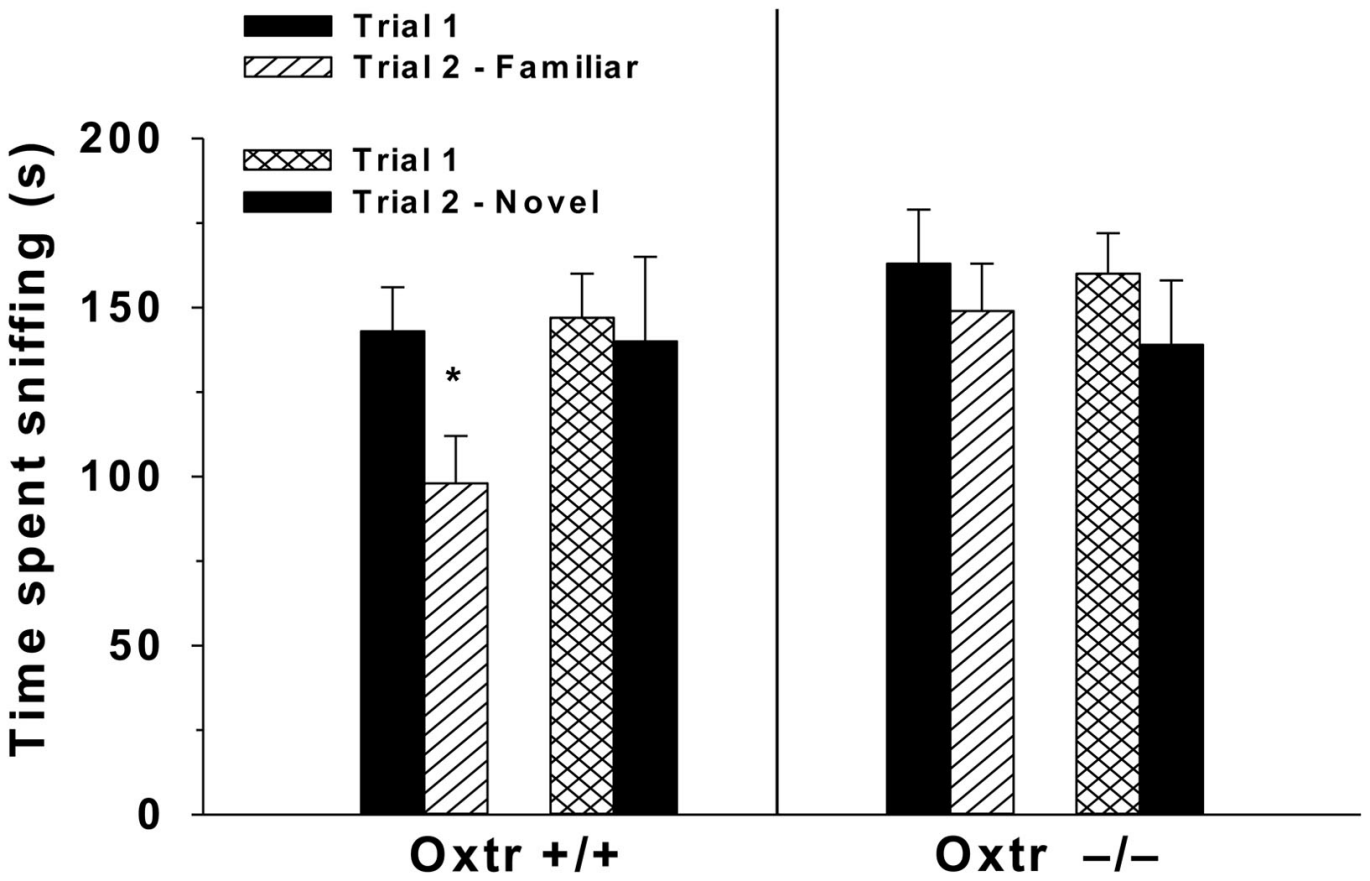
**Oxtr−/− mice have impaired social recognition**. In a two-trial discrimination task performed over 2 weeks, Oxtr+/+ (*n* = 8) and Oxtr−/− males (*n* = 8) were exposed to overiectomized BALB/C female mice during trial 1, and then 30 min later during trial 2 they were exposed to a familiar female on week 1. During week 2 of testing after trial 1, mice were exposed to a novel female during trial 2. Oxtr−/− mice fail to discriminate between the familiar and novel female spending approximately equal amounts of time sniffing both; compared to Oxtr+/+ mice that spend more time sniffing the novel female. Reprinted with permission from Endocrinology, Lee et al. (153).

The use of genetic tools, including Oxt−/− and Oxtr−/− mice have significantly contributed to our understanding of the role of Oxt in the social deficits observed in patients with schizophrenia. Male Oxt−/− and Oxtr−/− mice fail to develop social recognition memory, in essence having social amnesia (31, 32, 128, 153) (Figure 2). Further, an injection of Oxt into the MeA of Oxt−/− mice is able to restore social recognition (33, 154). These deficits in social memory are not specific to males, as female Oxt−/− mice do not show a normal Bruce effect (155, 156). Oxtr−/− mice also display behaviors similar to the negative symptoms of schizophrenia across multiple testing scenarios. In a visible burrow system, which provides a more natural habitat for rodents, Oxtr−/− mice have reductions in social interaction behaviors, spending more time alone and self-grooming than controls (129, 130). In a three-chamber test for sociability Oxtr−/− mice display increases in social withdrawal (129, 130) and in a social proximity test they display reductions in the frequency of nose-to-nose and nose-to-anogenital behaviors (129, 130). These data suggest that a functional Oxt system is necessary for normal social interactions, and that dysregulation of Oxt in schizophrenia could contribute to some of the negative symptoms.

Research also suggests that Oxt is important for other social behaviors, such as aggression and maternal behavior. Some studies have reported increases in violent behaviors in schizophrenic patients; however, it remains unclear whether this is a symptom of schizophrenia or rather co-morbid disorders (80, 81). Oxt−/− and Oxtr−/− mice have increases in aggressive behavior, and given the dysregulation of the Oxt system in schizophrenia, a functional Oxt system could be important for normal aggressive behavior (32, 90, 131, 153, 157, 158). Specifically, male Oxt−/− mice have heightened aggression when born to null mutant dams, but not when they are born to heterozygous dams (131, 157). Oxtr−/− mice also have heightened intermale aggression, but Oxtr FB/FB do not (32, 90, 153, 158). These data suggest that Oxt exposure during development may have persistent effects on aggressive behavior. Therefore, it could be that developmental Oxt contributes to the etiology of schizophrenia; however, more research is needed before such a claim can be made.

While there are no reported deficits in maternal behavior in patients with schizophrenia, the cognitive impairments associated with schizophrenia may lead to reductions in the ability to acquire necessary parenting skills (159–161). In animal models of schizophrenia, evidence suggests that decreases in maternal behaviors result in the development of behaviors similar to those found in other animal models of schizophrenia (83, 125, 126, 136, 162). Oxtr−/− and Oxtr FB/FB display deficits in the initiation of maternal behavior (32, 132, 133) and Oxt−/− mice pups emit fewer ultrasonic vocalizations when separated from nest; all of which suggest that Oxt contributes to social behavior in rodents (33, 131).

### Impaired cognition

The Oxt system may also be important to the cognitive dysfunctions associated with schizophrenia. One endophenotype of schizophrenia is impaired sensorimotor gating, i.e., the inability to “filter or gate” information (163, 164). Across species, sensorimotor gating can be measured using PPI of the startle reflex. The startle reflex is a defensive response to an abrupt, relatively intense stimuli (165). The neural circuitry that underlies PPI is known as the cortico-striato-pallido-pontine (CSPP) circuit (166). In humans, PPI is measured using electromyographic recordings from eye blink responses (167). In rodents, it is measured using the whole body flinch reflex of an animal to the startle stimulus (168). Patients with schizophrenia not only have reduced PPI but also show less habituation of the startle reflex compared to controls (169). In Brown Norway rats, which have a naturally low PPI, Oxt but not its structural analog carbetocin, is able to significantly increase PPI (170).

**Figure 3:**
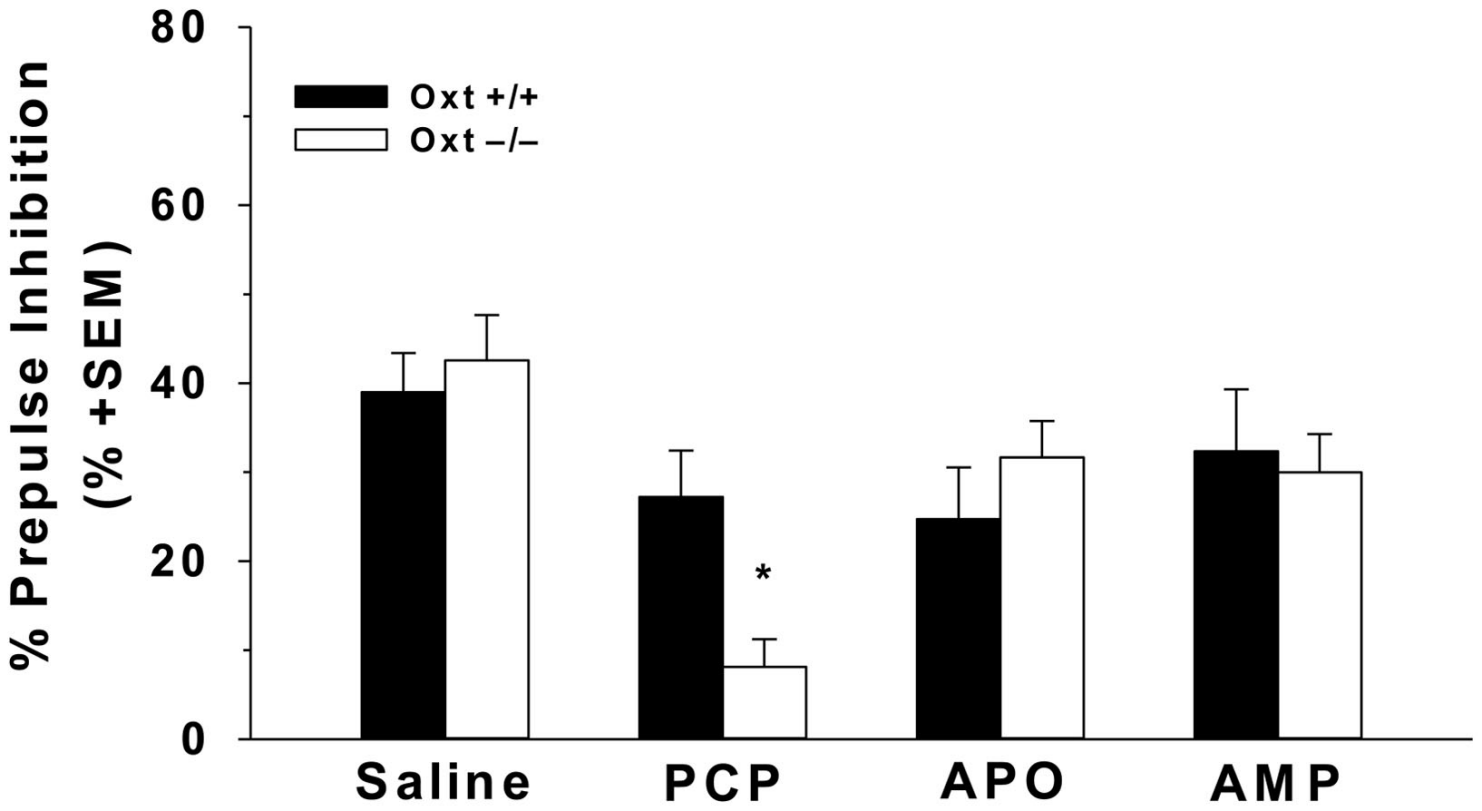
**Oxt−/− mice have greater PCP-induced deficits in sensorimotor gating**. The acoustic startle of male Oxt+/+ (*n* = 12) and Oxt−/− mice (*n* = 8) was measured using the whole body reflex flinch in reaction to a startle tone using startle chambers (SR-LAB; San Diego Instruments, San Diego, CA, USA). Mice were administered either an i.p. injection of 10 mg/kg AMP and APO, a subcutaneous injection 6 mg/kg PCP, or an equivalent volume of 0.9% saline as a control 15 min prior to testing. Testing session consisted of 60 trials, including no stimulus trials, pulse-alone trials, and prepulse + pulse trials. The testing sessions began and ended with the presentation of five 120 db pulse-alone tones. The middle 50 trials consisted of: 10 no pulse tones trials, 30 prepulse + pulse trials at 3, 6, and 12 db above background, and 10 pulse-alone tones at 120 db. A repeated measures design was used with each animal receiving 0.9% saline, AMP, APO, and PCP, with a minimum of 3 days between each trial. Oxt−/− mice display greater reductions in the average percent PPI across three prepulse levels (3, 6, and 12 db above background) following an injection of PCP compared to Oxt+/+ mice. There were no genotypic differences in PPI following injection of AMP or APO. Adapted and reprinted with permission from Macmillan Publishers Ltd: Molecular Psychiatry, Caldwell et al. (173).

Stress during the perinatal period may contribute to deficits in PPI, though there have been contradicting reports, with one group reporting deficits in PPI and another group finding no changes in PPI, some data suggests that early life stress reduces PPI levels in adulthood (162, 171). Changes in the Oxt system have been reported following early life stress, and may have an impact on perinatal stress-induced reductions in PPI, though more research is necessary (83, 122, 123). Further, in models that pharmacologically disrupt PPI, exogenous Oxt is known to reverse these deficits (170). Specifically, in rats, subcutaneous Oxt injections are able to restore deficits in PPI induced by AMP, an indirect dopamine agonist, and dizocipline (MK-801), a specific NMDA receptor antagonist, but not apomorphine (APO), a direct dopamine agonist (172). Finally, genetic disruptions in the Oxt system suggest that a lack of endogenous Oxt appears to be important in the regulation of PPI, as Oxt−/− mice have increased PCP-induced deficits in PPI (173) (Figure 3). This further suggests that the effects of endogenous Oxt on PPI may be specific to the glutamatergic system.

Oxt is likely to also contribute to the cognitive deficits associated with schizophrenia, such as impaired spatial memory and cognitive flexibility (3, 174). Similar to the cognitive deficits found in schizophrenia, Oxtr−/− mice display reduced cognitive flexibility, as measured by an inability to alter their behavior during the reversal phase of a *t*-maze task (175). Since the Oxtr is abundant in the hippocampus of mice, it may be important for memory (176). However, there are divergent reports of Oxt’s effects on spatial memory, suggesting that Oxt may have brain region-specific effects (38, 177). *In vitro*, hippocampal slices treated with Oxt are able to maintain long-term potentiation longer than untreated slices (38). In mouse dams, a central injection of Oxt is able to improve reference memory on a radial arm maze, but does not affect their short-term memory during acquisition, suggesting that Oxt only improves long-term spatial memory (38). As Oxt can improve anxiety in virgin mice when administered to the Amg or VMH, the effects of Oxt on reference memory may be due to its actions in these brain regions. However, there was no effect on their open-field activity, which suggests direct action on hippocampal neurons (38). Further, dams that receive an intracerebroventricular (i.c.v.) injection of an Oxt antagonist have reductions in reference memory compared to controls (38). But, in rats, Oxt injections into the nucleus basalis of Meynert (NBM) impair spatial memory, as measured by a Morris water maze, while an Oxtr antagonist injected into the NBM facilitates spatial memory (177). Given that disruptions in Oxt signaling appear to contribute to multiple aspects of cognition, and that Oxt may affect memory formation, it is plausible that Oxt may play a role in the cognitive deficits associated with schizophrenia.

The effects of Oxt dysregulation and Oxt treatment on the cognitive dysfunction found in patients with schizophrenia are poorly understood. Studies in both humans and animal models suggest that a functional Oxt system is required for normal sensorimotor gating and cognitive flexibility. The effects Oxt on sensorimotor gating may be specific to the glutamatergic system (172, 173), with mice lacking the obligatory NMDA receptor 1 subunit having impaired PPI (178). Unfortunately, as previously discussed, how these two systems interact remains unclear. The Oxt system is coupled to phospholipase c-β1 (PLC-β1) and glutamate is known to regulate PLC-β1 (36, 179–182). Abnormal expression patterns of PLC-β1 are found in patients with schizophrenia (183, 184). Further, studies using PLC-β1 knockout (PLCβ1−/−) mice find impaired PPI and deficits in working memory (185, 186). Therefore, the PLC-β1 may reflect a point of convergence for the Oxt and glutamate systems in the regulation of sensorimotor gating.

The effects of Oxt treatment on spatial learning are also ambiguous. While research suggests that Oxt in the hippocampus facilitates learning, it impairs memory when injected to the NBM (38, 177). However, while neuronal deficits in the hippocampus have been found, no reductions in neuronal density have been observed in the NBM in patients with schizophrenia (187). So, it is not clear whether or not this brain region is important to the pathophysiology of schizophrenia. In addition to Oxt’s effects on the cognitive deficits, it may also play a role in the positive symptoms associated with schizophrenia.

### Neuromotor abnormalities

**Figure 4:**
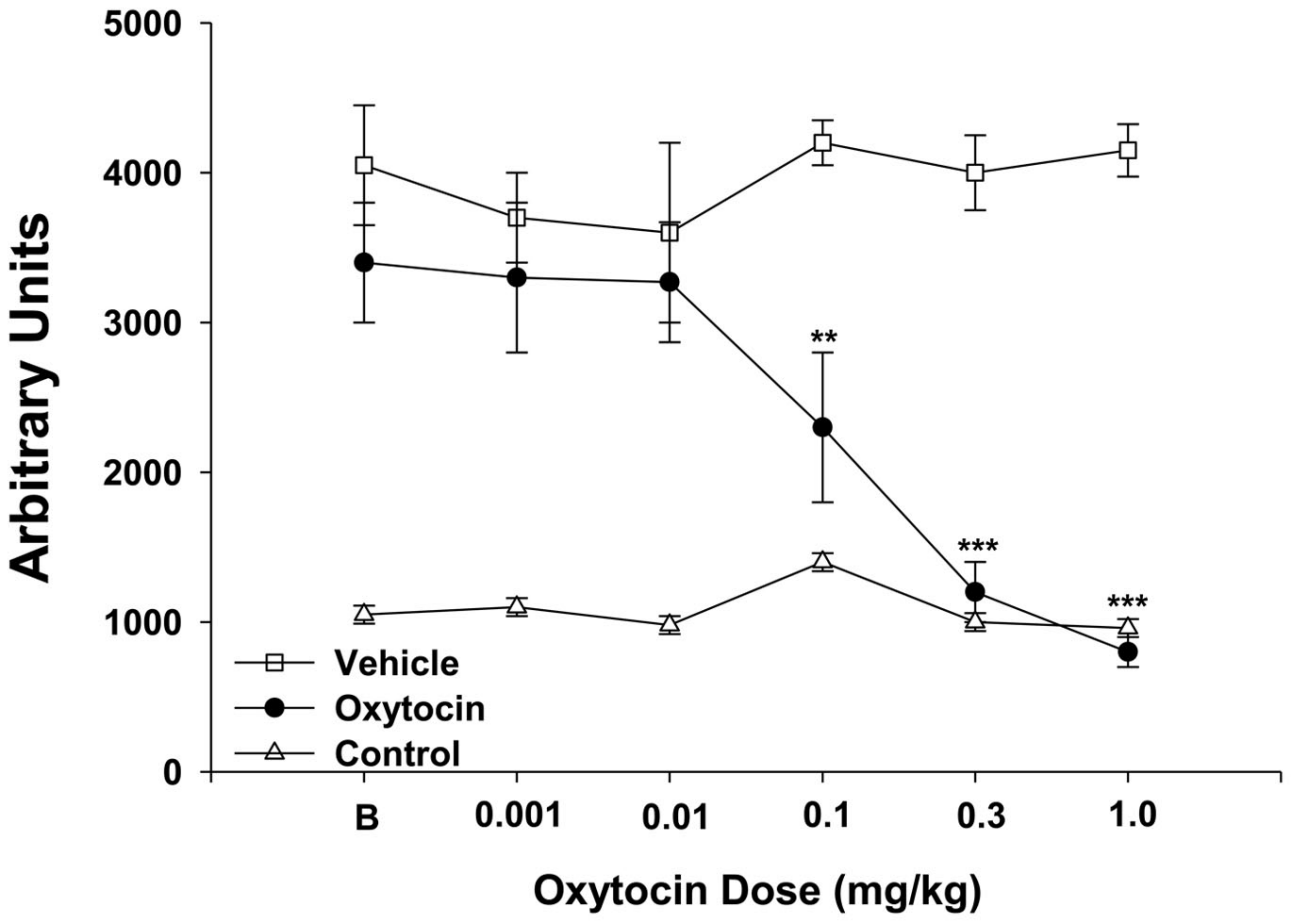
**Oxt dose-dependently decreases locomotor activity in self-administering methamphetamine rats**. Oxt (*n* = 5) was administered IP in ascending doses (0.001, 0.01, 0.1, 03, 1 mg/kg) over five consecutive days and equivalent amounts of vehicle (*n* = 5) were administered. Only the animals treated with Oxt or vehicle self-administered methamphetamine, and the control (*n* = 8) was used to determine baseline levels of locomotor activity. B = baseline day before oxytocin testing began. **p < 0.01 and ***p < 0.001. There was no difference between rats treated with Oxt compared to the control group at 0.3 and 1 mg/kg Oxt dose. All other comparisons between Oxt treatment and the control group and the vehicle treatment and control groups were significant. Data are shown as mean ± SEM. Adapted and reprinted with permission from Elsevier: Carson et al. (34).

In animal models, psychotic symptoms similar to the positive symptoms of schizophrenia can be manifested in rodents by treatment with dopamine agonists and NMDA receptor antagonists, which cause hyperlocomotor activity. While hyperlocomotor activity does not have direct face validity for the positive symptoms of schizophrenia, it does have construct validity as ­psychotomimetics cause similar neurotransmitter activity in animal models as is found in human ­schizophrenic patients. However, the behavioral effects are not necessarily similar; though some suggest that hyperlocomotor activity is comparable to some positive symptoms such as grossly disorganized behavior and psychomotor agitation (188, 189). Further, established antipsychotics, which reduce positive symptoms of ­schizophrenia, consistently reduce the hyperactivity associated with pharmacological agents such as AMP, cocaine, ketamine, and PCP. The antipsychotic efficacy of Oxt is supported by pharmacological manipulations that induce aspects of schizophrenia. During studies on addiction, Oxt decreases drug-induced hyperlocomotor activity (34, 35) while ­pretreatment with Oxt is able to attenuate the ­hyperlocomotor activity caused by cocaine, an indirect dopamine agonist (35) (Figure 4). In another study, which examined the effects of Oxt on addiction, i.c.v. injections of Oxt reduce ­methamphetamine-induced increases in locomotor activity (34). Other research also suggests that Oxt and the ­glutamatergic system may interact to affect the positive symptoms associated with schizophrenia. In addition to its behavioral effects, PCP induces the excessive release of glutamate within the medial prefrontal cortex (mPFC), which when blocked, suppresses hyperlocomotion (190, 191). Oxt has been found to reduce the PCP-induced symptoms associated with psychosis (173), as well as suppress glutamate release within the mPFC (192). Therefore, Oxt could suppress the hypofunction of glutamate specifically within the mPFC to protect against PCP-induced symptoms of psychosis. Genetic disruptions of the Oxt system also provide evidence that endogenous Oxt may affect locomotor activity, as there is hyperlocomotor activity in infant Oxtr−/− mice; however, this effect is not persistent (32).

## Oxytocin and the Pharmacology of Schizophrenia

Oxt is known to interact with several other neurotransmitter systems that are important in the etiology and treatment of schizophrenia, such as GABA and 5-HT (193). During parturition, Oxt has been found to modulate GABAergic inhibition in rodent models for autism spectrum disorder (ASD) (194). Given that ASDs and schizophrenia share similar endophenotypes, Oxt may also modulate GABA signaling in schizophrenic patients as well. However, further research is necessary to elucidate the role of the interactions of the Oxt system and GABAergic system to the symptomology of schizophrenia. Oxt and 5-HT are known to modulate one another, and both are important for numerous social behaviors and mood (195–197). Specifically, Oxt may exert anxiolytic effects via Oxtr activation in 5-HT neurons (195). Current atypical antipsychotics may provide further evidence for the interactions between the Oxt and 5-HT systems and schizophrenia.

Some of the currently used atypical antipsychotics are known to interact with the Oxt system. The atypical antipsychotics, amperozide and clozapine, increase plasma levels of Oxt, but the typical antipsychotic haloperidol does not (198). Amperozide and clozapine are both a 5-HT_2A_ antagonists, and to lesser extent D2 antagonists, that are reported to decrease both the negative and positive symptoms associated with schizophrenia (199–202). Whereas, the D2 specific antagonist, haloperidol, only appears to alleviate positive symptoms of schizophrenia (203, 204). Further, some atypical antipsychotics cause activation of Oxt cells as measured by cFos ir. Clozapine increases cFos activation in Oxt cells in the PVN, but again, haloperidol treatment does not (205). Similar to the effects of Oxt, in rodents, clozapine attenuates the reduction of cognitive flexibility caused by the sub-chronic PCP treatment (206), and is able to restore normal levels of PPI to brown Norway rats (207). This evidence further supports a role of the Oxt in the cognitive deficits found in schizophrenic patients. In humans, clozapine attenuates both the negative symptoms and cognitive dysfunctions found in patients with schizophrenia (208–212). Therefore, the ability to reduce the social and cognitive deficits may be associated with the ability of clozapine to increase Oxt levels. Further, the specific interactions between the Oxt and serotonergic systems may be important to the social and cognitive deficits found in patients with schizophrenia. However, additional research is necessary to assess how Oxt may affect the symptom domains associated with schizophrenia through its interactions with other neurotransmitters systems.

## Conclusion

Given the importance of the Oxt system to the modulation of social behaviors, it is not surprising that across animal models of schizophrenia, Oxt has been implicated in the negative symptoms and deficits in social cognition. Data suggests that developmental, drug induced, and genetic disruptions in the Oxt system lead to the symptoms associated with the negative symptoms observed in schizophrenic patients. However, further research is needed to elucidate the specific mechanisms whereby Oxt exerts these effects. Human and animal models also suggest that research is needed to determine if Oxt can work as a therapeutic agent to improve the social behavior deficits observed in patients with schizophrenia. Oxt also appears to be a contributor to the cognitive and positive symptom domains of schizophrenia; though much more work in this area is needed. While Oxt does not “cause” schizophrenia, its putative impact to all three symptom domains suggests that it may be an important player to the etiology, and perhaps even an effective treatment, of schizophrenia. Using animal models, future research will need to focus on elucidating of the mechanisms of Oxt dysregulation and the interactions between Oxt and other neurotransmitter systems that may contribute to the symptoms associated with schizophrenia.

## Conflict of Interest Statement

The authors declare that the research was conducted in the absence of any commercial or financial relationships that could be construed as a potential conflict of interest.
